# The Societal Influences and Quality of Life Among Healthcare Team Members During the COVID-19 Pandemic

**DOI:** 10.3389/fpsyt.2021.706443

**Published:** 2021-10-11

**Authors:** Wei-Tsung Kao, Su-Ting Hsu, Frank Huang-Chih Chou, Li-Shiu Chou, Kuan-Ying Hsieh, Dian-Jeng Li, Guei-Ging Lin, Pay-Jen Wu, Wei-Jen Chen, Joh-Jong Huang

**Affiliations:** ^1^Kaohsiung Municipal Kai-Syuan Psychiatric Hospital, Kaohsiung, Taiwan; ^2^Department of Sports, Health and Leisure and Graduate Institute of Sports, Health and Leisure, Cheng Shiu University, Kaohsiung, Taiwan; ^3^Graduate Institute of Counseling Psychology and Rehabilitation Counseling, National Kaohsiung Normal University, Kaohsiung, Taiwan; ^4^College of Medicine, Graduate Institute of Medicine, Kaohsiung Medical University, Kaohsiung, Taiwan; ^5^Department of Nursing, Meiho University, Pingtung, Taiwan; ^6^Department of Family Medicine, Kaohsiung Medical University Hospital, Kaohsiung, Taiwan; ^7^Department of Health, Kaohsiung City Government, Kaohsiung, Taiwan

**Keywords:** disaster-related psychological screening test (DRPST), societal influences survey questionnaire (SISQ), short form-12 items (SF-12), quality of life, corona virus infection disease 2019 (COVID-19), structural equation model (SEM)

## Abstract

**Background:** The coronavirus infection disease 2019 (COVID-19) pandemic is likely to put healthcare professionals across the world in an unprecedented situation.

**Methods:** A total of 683 healthcare workers were recruited in this study. Short form*-*12 items (SF-12), Societal Influences Survey Questionnaire (SISQ), and Disaster-Related Psychological Screening Test (DRPST) were used to survey participants. Multiple linear regression and structural equation model (SEM) were used to explore the possible factors to the societal influences and quality of life.

**Results:** After multiple linear regression analysis, female, older, more education years, married, regular intake, and posttraumatic stress disorder (PTSD) frequency had positive association with SISQ. To physical component summary (PCS) of SF-12, chronic illness, sleep score, PTSD frequency, and social distance had negative association, and exercise habits had positive association. A mental component summary (MCS) value of SF-12, age, participate in social activities, and social information had positive association, and PTSD frequency, sleep score, social anxiety, and depression had negative association. Under SEM analysis, PTSD had positive influence on SISQ. Sleep score and MCS value had negative influences on SISQ. PTSD severity, older age, sleep score, smoking, and nursing staff had negative influences on PCS value. Young age, PTSD frequency, sleep score, and depression had negative influences on MCS value.

**Conclusion:** Healthcare team members with severe PTSD symptoms suffered more societal influences. Relative to PTSD severity, PTSD frequency was more important to the quality of life. Members of older age who frequently participate in clubs, volunteers, or charity activities had better mental life quality.

## Background

The COVID-19 pandemic is likely to put healthcare professionals across the world in an unprecedented situation, having to make impossible decisions and work under extreme pressures (1). Among the healthcare workers also, they are involved directly in handling patients and are at greater risk than others. The reasons for such adverse psychological outcomes in them range from excessive workload/work hours, inadequate personal protective equipment, over-enthusiastic media news, and feeling inadequately supported (2, 3).

Another important reason for such psychological impact is the infection risk among medical staff. The sudden reversal of role from a healthcare worker to a patient might lead to frustration, helplessness, adjustment issues, stigma, and fear of discrimination in the medical staff (4). Despite the low mortality rate of 2%, the COVID-19 virus has a high transmission rate and the number of deaths is higher than that caused by severe acute respiratory syndrome (SARS) and Middle East respiratory syndrome (MERS) combined (5). The psychological impact of COVID-19 and other pandemic among healthcare team members is an important issue to us.

In the past, there were several studies (6–8) about the association between mental health and biological disasters (like SARS, COVID-19) among healthcare members. A previous study reported that mental health problems were associated with social interaction and the ability to cope with COVID-19 among the general public in Taiwan (9).

In the face of such biological disaster public health incidents like COVID-19, medical staff had massive stress on physical and mental health (10). And past studies (11–15) on the psychological impact of the SARS outbreak in Taiwan have focused on the psychiatric morbidity of medical professionals. In Taiwan's past experience in dealing with SARS, a study by a regional teaching hospital (11) showed that 17.3% of the medical staff involved in the care of SARS patients had obvious psychiatric symptoms. A follow-up study (12) after 3 years found that the medical staff with psychiatric symptoms showed that these symptoms were related to the stress of daily life and were less related to the SARS crisis. Compared with nurses, doctors have a higher ratio of physical symptoms, which indicates that different professionals suffered from different stress of mental health.

For social distance, many studies (16–20) had showed that the effect of social distance on COVID-19 was very important. Increasing social distance can significantly reduce the infection rate of COVID-19 (19). In a past study (21), a massive impact of COVID-19 and previous epidemics like SARS on social activities was found. As we know, social distance during the COVID-19 pandemic is necessary to everyone. On the other hand, the interference on social activities may have substantial mental health impacts (22).

For social anxiety, a study (23) indicated a high proportion of anxiety symptoms (35.1%) among the Chinese in China from the online data during the COVID-19 pandemic. Another study (24) also indicated higher levels of anxiety were correlated with social isolation and quarantine during the SARS pandemic. Past study (18) showed that high social anxiety predicted higher perceptions of illness and lower judgments of trustworthiness. Another study (25) found that anxiety was associated with stress and reduced sleep quality, and the combination of anxiety and stress reduced the positive effects of social capital on sleep quality. A Taiwanese study demonstrates that excessive anxiety because of COVID-19 is associated with lower subjective psychological well-being (26).

One past study (27) showed that social media related information spreading can strongly affect people's behavior and alter the effectiveness of the countermeasures deployed by governments. The social information may affect the other domains of societal influence. The significant association between receiving information about COVID-19 from more sources and greater confidence was found in healthcare team members (28).

Social adaptation implies the intention of subjects to change their behavior and protect themselves to prevent COVID-19 infection. Several studies (29–33) showed that there were associations between protective behavior and the COVID-19 pandemic. There may be some factors that affect social adaptation of healthcare team members. Let them be willing to change their behavior and reduce the risk of being infected during the COVID-19 pandemic.

For the quality of life among healthcare team members during the COVID-19 pandemic, a past study (34) showed that the quality of life among medical staff was decreased. In a pandemic, healthcare workers face greater risk of infection and undertake higher work intensity as compared with the general population.

About the related factors of quality of life among healthcare team members, physical activity, and higher health literacy were found to protect against anxiety and depression and were associated with higher health related quality of life (35). Unexpectedly, smoking and drinking were also found to be coping behaviors. It is important to have strategic approaches that protect healthcare team members' mental health and health related quality of life. Measuring the healthcare workers' risk perception of the COVID-19 and the relevant influential factors can provide the service providers, health policy makers, as well as the health and hygiene instructors with great insights on facilitation of the behaviors aiming at self-effectiveness in improving community health and selecting the best solutions for minimizing the risks arising from this disease. The main aims of this study were (i) to investigate the possible factors to the societal influences and quality of life and (ii) to explore interrelationships and underlying mechanisms between societal influences, mental life quality, physical life quality, and related factors among the healthcare team members in a large psychiatric hospital during the coronavirus infection disease 2019 pandemic in Taiwan.

## Methods

### Subjects

A cross-sectional investigation was used in the present study. A total of 716 participants were collected from July 2020 to September 2020 at a psychiatric teaching hospital in southern Taiwan. Only 33 subjects did not complete questionnaires. A satisfactory response rate of 95.3% was thus obtained. Thus, this study consisted of 683 health care workers, including 44 physicians, 283 nurses, and 356 other hospital healthcare workers. The inclusion criteria were: (1) participants who worked at a large psychiatric hospital in southern Taiwan, (2) could understand the objective of the study and follow the instructions from research assistants, (3) aged between 20 and 65 years, and (4) informed consent was obtained from all subjects before filling in the questionnaire. Data with missing values or from those who could not complete the questionnaire were excluded. This study was approved by the Institutional Review Board of KSPH (KSPH-2020-03) and conducted according to the current revision of national legal requirements (Human Subjects Research Act, Taiwan).

Societal influences, quality of life, posttraumatic stress disorder (PTSD) symptoms, levels of depression, sleep disturbance, and related demographic variables were collected through self-reported questionnaires.

*T*-test and Chi-square test were performed on the demographic variables and questionnaires between nursing staff and non-nursing staff. Multiple linear regression analyses were conducted to ascertain whether the independent factors were associated with dependent variables, including societal influences and quality of life (MCS and PCS). Structural equation model (SEM) was used to explore the possible factors to the societal influences and quality of life among healthcare team members in a psychiatric hospital. We also try to find out the associations of other latent variables and their relationship to PTSD scales (PTSD severity score and PTSD frequency score), level of depression, and sleep disturbance by path analysis.

### Measures

#### Demographic Data

Data was recorded with the participants' age, educational years, marital status, gender, religion, types of work in the hospital, smoking (yes: currently smoking more than five cigarettes a day or no), alcohol use (≥3 times per week or not), Exercises habits (≥3 days per week or not), participation in social activities (participate in clubs, volunteer or charity activities or not), regular intake (three or four meals a day, ≥5 days per week or not), chronic physical illness within 1 year, and history of hypertension and diabetes. All of the demographic information was identified as categorical variables except age and educational years were continuous variables.

#### Societal Influences Survey Questionnaire (SISQ)

The Societal Influences Survey Questionnaire (SISQ) was constructed to measure the psychological and social impact on individuals during the COVID-19 pandemic. With acceptable validity and reliability, the 15-item SISQ contains five categories of assessment: social distance, social anxiety, social desirability, social information, and social adaptation (36). Ten of the questions were selected in the current study with four domains: social distance, social anxiety, social information, and social adaptation. Cronbach's alpha of 10-item SISQ was 0.817 in the study. The overall internal consistency coefficient (Cronbach's α) of the 15-item questions SISQ was 0.74 in our past study (36). And Cronbach's alphas were 0.76/0.70/0.56/0.54 among social distance/social anxiety/social information/social adaptation four domains. We adapted 10-item SISQ in our study. Each question was scored on a 4-point Likert scale, with scores ranging from 1 (*never*) to 4 (*often*). Higher total scores of each domain (social distance, social anxiety, social information, and social adaptation) indicated higher compliance to maintain social distance, higher level of anxiety due to COVID-19, more desire to seek related information, and more awareness of progress of the pandemic overseas, respectively. Our past study (36) demonstrated that the SISQ has acceptable reliability, with Cronbach's alphas ranging between 0.57 and 0.76. The SISQ accounted for 58.86% and satisfied the requirement of Kaiser–Mayer–Olkin values (0.78) and significant Bartlett's Test of sphericity. Moreover, the confirmatory factor analysis fit indices also indicated the adequacy of the model.

#### Short Form-12 Items Health Survey (SF-12)

The 12-item Short Form Survey version 2 (SF-12v2) is based on scoring coefficients derived from version 1 of the SF-36. It was developed to rapidly estimate general health status and has since been well-validated (37). The Short form*-*12 items health survey (SF-12) (38) is one of the most commonly used health-related quality of life (HRQoL) questionnaires, and it has become widely used in community-based health surveys and physical and mental illness outcome assessments due to its brevity and psychometric performance (39, 40). A recent study (41) showed that PCS and MCS demonstrated high internal consistency (Cronbach's α–PCS: 0.87, MCS: 0.86) and good and moderate test-retest validity, respectively (intraclass correlation coefficient: PCS: 0.79, MCS: 0.59). A previous study (42) reported acceptable to good levels of reliability for PCS internal consistency coefficient (ICC) (Cronbach's α = 0.78) and MCS ICC (Cronbach's α = 0.60).

These items were graded on a 5-point Likert scale, with scores ranging from 1 (*not at all*) to 5 (*extremely*). A higher score represented a higher level of interference. And the questionnaire contained two components which were mental component summary (MCS) and physical component summary (PCS). So the score of SF-12v2 was divided into “PCS value of SF-12” and “MCS value of SF-12.”

#### Depression Score

*Depression Scales From the Disaster-Related Psychological Screening Test*. The level of depression was measured using three questions from the Disaster-Related Psychological Screening Test (DRPST). The DRPST has been shown to be reliable and well-validated to rapidly screen for major depressive disorder or posttraumatic stress disorder (PTSD) after a disaster (43, 44). Three items were used to estimate the status of depressed mood, fatigue or loss of energy, and worthlessness which had persisted for more than 2 weeks in the recent 1 month. Each item was rated on a 2-point Likert scale, with scores ranging from 0 (*no*) to 1 (*yes*). In a past study (44), a score of 2 or more on the depression scale of DRPST was used to define positive cases of major depressive disorder, giving a sensitivity of 92.1%, specificity of 98.3%, positive predictive value of 83.3%, and negative predictive value of 99.3%. Higher total scores of the three items indicated higher levels of depression. Details of the questionnaire are listed in Supplementary Table 1.

#### Sleep Score

*Sleep Disturbance Scales From the Pittsburgh Sleep Quality Index*. The Pittsburgh Sleep Quality Index (PSQI) was initially developed to measure the sleep quality in clinical populations, and it has been shown to have good validity and reliability (45). A global PSQI score > 5 yielded a diagnostic sensitivity of 89.6% and specificity of 86.5% (kappa = 0.75, *p* ≤ 0.001) in distinguishing good and poor sleepers. And it indicated a reliability coefficient (Cronbach's α) of 0.83. Four items selected from the PSQI were used to estimate the level of sleep disturbance: difficulty to fall asleep, waking up in the middle of the night, subjective sleep quality, and enthusiasm in the recent 1 month (Supplementary Table 1). Each item was rated on a 4-point Likert scale, with scores ranging from 1 to 4. Higher total scores of the four items indicated more severe sleep disturbance.

#### Post-Traumatic Stress Disorder (PTSD) Symptoms

*Post-Traumatic Stress Disorder (PTSD) Scales From the Disaster-Related Psychological Screening Test*. The levels of PTSD symptoms were measured using eight questions from the Disaster-Related Psychological Screening Test (DRPST). The DRPST has been shown to be reliable and well-validated to rapidly screen for major depressive disorder or post-traumatic stress disorder (PTSD) after a disaster (43, 44). A score of 3 or more on the PTSD scale of DRPST was used to define the positive cases; this resulted in the most appropriate sensitivity (97.8%) and specificity (96.6%), a positive predictive value of 76.3%, and a negative predictive value of 99.8%. The PTSD scales divided into two components: PTSD severity score and PTSD frequency score. Eight items of PTSD severity and PTSD frequency were used to estimate the status of PTSD in the recent 1 month. Each item was rated on a 5-point Likert scale, with scores ranging from 1 (*PTSD severity: no distress; PTSD frequency: never*) to 5 (*PTSD severity: extremely distress; PTSD frequency: every day*). Higher total scores of the eight items indicated higher levels of PTSD severity and PTSD frequency. Details of the questionnaire are listed in Supplementary Table 1.

### Statistical Analysis

Because nursing staff accounted for a large proportion of the overall number of nursing staff in the hospital, and in our study, 283 of all 683 cases were nursing staff, accounting for 41.5%, and nursing staff often had to work in shifts. The work style is different, so we divide all cases into two groups of nursing staff and non-care staff. *T*-test and Chi-square test were performed on the demographic variables and questionnaires between nursing staff and non-nursing staff. Marital status was transformed into a dichotomous variable as “married” (married and remarried) or “unmarried” (single, widowed, cohabiting, and divorced) (Supplementary Table 2). Histories of chronic illness were also transformed into dichotomous variables as “yes” or “no” (Supplementary Table 3).

The data were analyzed using the IBM SPSS Statistics for Windows, Version 21.0 (IBM Corp. Released 2012. Armonk, NY: IBM Corp). Baseline characteristics and the scores of questionnaires were compared using an independent *T*-test or the chi-square test. Multiple linear regression analyses were conducted to ascertain whether the independent factors were associated with dependent variables, including SISQ total score_10, PCS value of SF-12, and MCS value of SF-12. The alpha level was set at 0.05.

The normality of dependent variables was checked using the Kolmogorov-Smirnov test. Because non-normally distributed samples were identified with the significance of the test (*p* < 0.001), bootstrapping multiple linear regression with 1,000 bootstrap samples was used to verify the results from the stepwise multi-variate linear regression analysis. In the bootstrapping method, 95% confidence intervals were used to determine statistical significance, as this could qualify the stability of the regression coefficients and reduce the length of the confidence interval (46). When the 95% confidence interval of a regression coefficient did not contain zero, the variable was statistically significant. In addition, the number of bootstrap samples was set as 1,000 to obtain sufficiently accurate 95% bootstrap percentile (47).

The AMOS 21.0 (SPSS, Chicago, IL, USA) was used to analyze the structural equation model (SEM). SEM uses the chi-square fit test to investigate the overall fit into the model; chi-squares resulting in *P* > 0.05 and an adjusted goodness-of-fit index >0.9 indicated that the model adequately describes the observed data. Root Mean Square Error of Approximation (RMSEA) is based on the non-centrality parameter. Good models have an RMSEA of 0.05 or less. Models whose RMSEA is 0.10 or less were necessary (48). Values for the SRMR range from 0 to 1.0 with well-fitting models obtaining values <0.05 (49, 50), however values as high as 0.08 are deemed acceptable (51).

Furthermore, we explore the possible factors to the societal influences and quality of life among healthcare team members in a psychiatric hospital by SEM. We also try to find out the associations of other latent variables and their relationship to quality of life, PTSD scales, depression, and sleep by path analysis.

## Results

A total of 716 participants were collected from July 2020 to September 2020 at a large psychiatric teaching hospital in southern Taiwan. Only 33 subjects did not complete questionnaires. A satisfactory response rate of 95.3% was thus obtained. And 33 subjects who did not complete questionnaires were older and had fewer education years than 683 subjects who completed questionnaires. In other demographic data (like gender, different healthcare professions, smoking, or not), there were no statistically significant differences. Thus, this study consisted of 683 health care workers, including 44 physicians, 283 nurses, and 356 other hospital health care workers.

### Demographic Data and Questionnaires Between Nursing Staff and Non-Nursing Staff

In Table 1, we used *T*-test and Chi-square test to compare the difference of demographic data and questionnaires between two groups (nursing staff group vs. non-nursing staff group). We found that non-nursing staff group had more male than nursing staff group (χ^2^ = 45.238, *P* < 0.001), non-nursing staff were older than nursing staff (*T* = 97.569, *P* < 0.001), more non-nursing staff were married than nursing staff (χ^2^ = 7.229, *P* = 0.007), more non-nursing staff had religion belief than nursing staff (χ^2^ = 7.127, *P* = 0.008), more non-nursing staff had smoking than nursing staff (χ^2^ = 13.593, *P* < 0.001), and more non-nursing staff had exercise habits (χ^2^ = 38.018, *P* < 0.001), regular intake (χ^2^ = 42.985, *P* < 0.001), and participate in social activities (χ^2^ = 28.989, *P* < 0.001) than nursing staff. More non-nursing staff had chronic physical illness than nursing staff (χ^2^ = 5.384, *P* = 0.020) within 1 year, more non-nursing staff had hypertension than nursing staff (χ^2^ = 4.752, *P* = 0.029), and more non-nursing staff had DM than nursing staff (χ^2^ = 5.958, *P* = 0.015). There were no significant statistically differences over alcohol use (χ^2^ = 0.032, *P* = 0.859) and education years (*T* = 2.689, *P* = 0.015) between two groups. In the questionnaires, nursing staff group had more social anxiety (*T* = 6.046, *P* = 0.014) and less social information (*T* = 3.894, *P* = 0.049) than non-nursing staff group. Nursing staff had more sleep disturbance than non-nursing staff under sleep score analysis (*T* = 9.281, *P* = 0.002). Nursing staff had lower score of MCS value than non-nursing staff among SF-12v2 analysis (*T* = 13.259, *P* < 0.001). There were no significant statistically differences over SISQ total score (*T* = 0.428, *P* = 0.513), social distance (*T* = 3.385, *P* = 0.066), social adaption (*T* = 0.093, *P* = 0.760), PCS value of SF-12v2 (*T* = 2.855, *P* = 0.092), depression score (*T* = 0.723, *P* = 0.396), PTSD frequency score (*T* = 0.021, *P* = 0.885), and PTSD severity score (*T* = 0.675, *P* = 0.412).

**Table 1:** The difference of demographic data and questionnaires between nursing staff and non-nursing staff.

	**Non-nursing staff**	**Nursing staff**	**χ^**2**^**	***T***	***P*-value**
	**(*n* = 400)**	**(*n* = 283)**			
Male	137 (34.3%)	33 (11.7%)	45.238		<0.001
Married	198 (49.6%)	111 (39.2%)	7.229		0.007
Had religion	273 (68.3%)	165 (58.3%)	7.127		0.008
Smoking	36 (9.0%)	6 (2.1%)	13.593		<0.001
Alcohol use	47 (11.75%)	32 (11.3%)	0.032		0.859
Exercises habits	279(69.8%)	131 (46.3%)	38.018		<0.001
Regular intake	357 (89.3%)	196 (69.3%)	42.985		<0.001
Participate in social activities	98 (24.5%)	24 (8.5%)	28.989		<0.001
Chronic physical illness within 1 year	86 (21.5%)	41 (14.5%)	5.384		0.020
Hypertension	28 (7.0%)	9 (3.2%)	4.752		0.029
Diabetes mellitus	17 (4.3%)	3 (1.1%)	5.958		0.015
Age	42.5 ± 10.8	34.9 ± 8.4		97.569	<0.001
Education years	16 ± 3	16.3 ± 1.7		2.689	0.101
Total score SISQ	27 ± 6.1	26.6 ± 6.4		0.428	0.513
Social distance	11.8 ± 3.1	11.4 ± 3		3.385	0.066
Social anxiety	4 ± 1.6	4.3 ± 1.7		6.046	0.014
Social information	5.1 ± 1.6	4.9 ± 1.6		3.894	0.049
Social adaptation	6 ± 1.9	6.1 ± 1.9		0.093	0.760
PCS value	52.1 ± 5.5	51.4 ± 6.1		2.855	0.092
MCS value	48.8 ± 9.7	46.2 ± 8.8		13.259	<0.001
Depression score	0.3 ± 0.8	0.4 ± 0.8		0.723	0.396
PTSD frequency score	2.6 ± 3.8	2.7 ± 3.7		0.021	0.885
PTSD severity score	2 ± 3.1	2.2 ± 3.4		0.675	0.412
Sleep score	4.9 ± 2.4	5.6 ± 2.9		9.281	0.002

*SISQ, Societal Influences Survey Questionnaire; PCS, physical component summary; MCS, mental component summary; PTSD, posttraumatic stress disorder*.

### Multiple Linear Regression for Possible Related Factors of Total SISQ Score

After multiple linear regression (Table 2), we found that female (β = 0.143; *P* < 0.001), older subjects (β = 0.113; *P* = 0.005), more education years (β = 0.103; *P* = 0.004), had marriage (β = 0.077; *P* = 0.049), regular intake (β = 0.109; *P* = 0.003), and PTSD frequency score (β = 0.313; *P* < 0.001) all had positive association with SISQ score. After verification with 1,000 bootstrapping multiple linear regressions, the significant related factors were the same as in multiple linear regression (Supplementary Table 4).

**Table 2:** Multiple linear regression for possible related factors of Total SISQ score.

**Variable**	**Beta**	***t***	**95% CI**		***P*-value**
			**Lower bound**	**Upper bound**	
Female	0.143	4.072	1.068	3.058	<0.001
Married	0.077	1.970	0.003	1.919	0.049
Regular intake	0.109	3.030	0.606	2.836	0.003
PTSD frequency score	0.313	8.867	0.402	0.630	<0.001
Age	0.113	2.847	0.021	0.112	0.005
Education years	0.103	2.854	0.079	0.429	0.004

*Dependent variable: Total score SISQ*.

### Multiple Linear Regressions for Possible Related Factors of PCS Value of SF-12

After multiple linear regression (Table 3), we found that chronic illness within 1 year (β = −0.160; *P* < 0.001), smoking (β = −0.076; *P* = 0.031), sleep score (β = −0.237; *P* < 0.001), MCS value of SF-12 (β = −0.151; *P* < 0.001), and PTSD frequency score (β = −0.200; *P* < 0.001) and social distance (β = −0.098; *P* = 0.007) all had negative association with PCS value of SF-12. On the other hand, exercise habits (β = 0.147; *P* < 0.001) had positive association with PCS value of SF-12. After verification with 1000 bootstrapping multiple linear regression, the significant related factors were the same as in multiple linear regression except smoking was excluded from bootstrapping methods (Supplementary Table 5).

**Table 3:** Multiple linear regression for possible related factors of PCS value of SF-12.

**Variable**	**Beta**	***t***	**95% CI**		***P*-value**
			**Lower**	**Upper**	
			**bound**	**bound**	
Chronic illness within 1 year	−0.160	−4.529	−3.414	−1.349	<0.001
Smoking	−0.076	−2.167	−3.493	−0.172	0.031
Exercises habits	0.147	4.125	0.906	2.553	<0.001
Sleep score	−0.237	−5.755	−0.689	−0.339	<0.001
MCS Value	−0.151	−3.548	−0.143	−0.041	<0.001
PTSD frequency score	−0.200	−4.750	−0.433	−0.180	<0.001
Social Distance	−0.098	−2.725	−0.319	−0.052	0.007

*Dependent variable: PCS value of SF-12*.

### Multiple Linear Regression for Possible Related Factors of MCS Value of SF-12

After multiple linear regression (Table 4), we found that age (β = 0.147; *P* < 0.001), participate in social activities (β = 0.067; *P* = 0.026), and social information (β = 0.105; *P* = 0.002) all had positive association with MCS value of SF-12. On the other hand, PTSD frequency score (β = −0.265; *P* < 0.001), sleep score (β = −0.211; *P* < 0.001), social anxiety (β = −0.193; *P* < 0.001), depression score (β = −0.232; *P* < 0.001), and PCS value of SF-12 (β = −0.093; *P* = 0.003) all had negative association with MCS value of SF-12. After verification with 1,000 bootstrapping multiple linear regression, the significant related factors were the same as in multiple linear regression (Supplementary Table 6).

**Table 4:** Multiple linear regression for possible related factors of MCS value of SF-12.

**Variable**	**Beta**	***t***	**95% CI**		***P*-value**
			**Lower**	**Upper**	
			**bound**	**bound**	
Age	0.147	4.728	0.077	0.186	<0.001
Participate in social activities	0.067	2.231	0.198	3.106	0.026
PTSD frequency score	−0.265	−7.213	−0.844	−0.483	<0.001
Sleep score	−0.211	−5.855	−1.000	−0.498	<0.001
Social Anxiety	−0.193	−5.415	−1.488	−0.696	<0.001
Social Information	0.105	3.110	0.228	1.009	0.002
Depression score	−0.232	−6.675	−3.629	−1.979	<0.001
PCS Value	−0.093	−3.003	−0.252	−0.053	0.003

*Dependent variable: MCS value of SF-12*.

### The Structural Equation Model (SEM) Showing Interrelationships Between Societal Influences Survey Questionnaire (SISQ), Mental Component Summary (MCS) Value, and Physical Component Summary (PCS) Value of Short Form-12 Items Health Survey (SF-12), PTSD, Age, Sleep Score, Depression Score, Nursing Staff, Gender, Education Years, and Smoking

**Figure 1:**
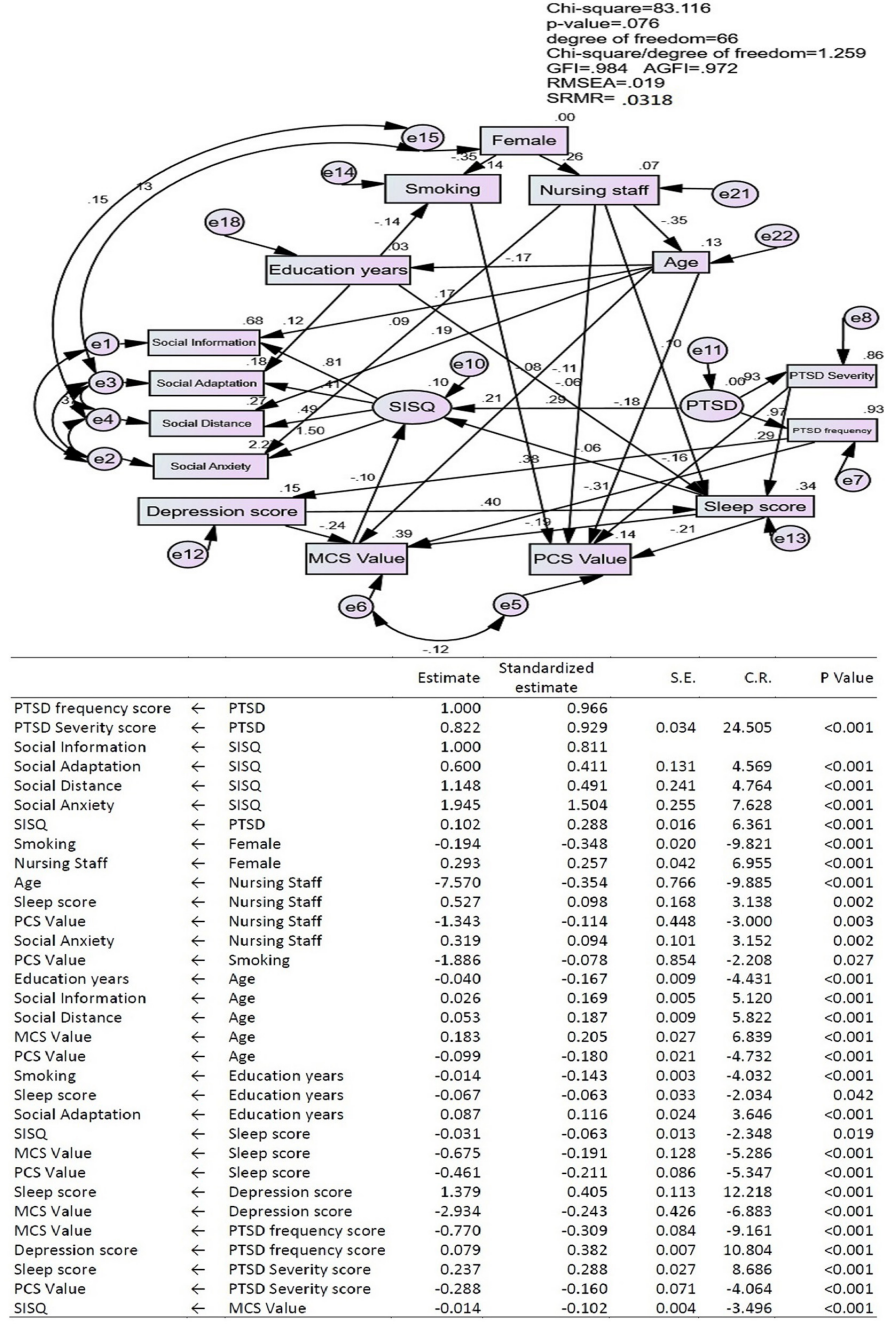
The conceptual model showing interrelationships between Societal Influences Survey Questionnaire (SISQ), mental component summary (MCS) value, and physical component summary (PCS) value of Short form-12 items health survey (SF-12v2), PTSD, Age, Sleep score, depression score, Nursing Staff, gender, education years, and Smoking.

We used Structural Equation Model (SEM) analysis to explore interrelationships between Societal Influences Survey Questionnaire (SISQ), mental component summary (MCS) value, and physical component summary (PCS) value of Short form-12 items health survey (SF-12), PTSD, age, sleep score, depression score, nursing staff, gender, education years, and smoking. In our SEM model, our *P*-value is 0.422 (>0.05), adjusted goodness-of-fit index (AGFI) is 0.977 (>0.9), RMSEA is 0.019 (<0.05), and SRMR is 0.0318 (<0.05) which indicated that our model is a good model and adequately describes the data in our study. In Figure 1, we showed that PTSD (two components: PTSD severity and PTSD frequency) had positive influence on SISQ (four components: social information, social adaption, social distance, and social anxiety). Otherwise, sleep score and MCS value both had negative influences on SISQ.

Age had a positive influence on social information and social distance. Education years had a positive influence on social adaption. Nursing staff had a positive influence on social anxiety.

PTSD severity had a positive influence on sleep score and a negative influence on PCS value. PTSD frequency had a positive influence on depression score and a negative influence on MCS value. Depression score and nursing staff had a positive influences on sleep score. Age had a positive influence on MCS value and a negative influence on PCS value. Sleep score and depression score both had negative influences on MCS value. Sleep score, smoking, and nursing staff all had negative influences on PCS value.

Female had a negative influence on smoking and a positive influence on nursing staff. Nursing staff had a negative influence on age. Age had a negative influence on education years. Education years had a negative influence on smoking.

## Discussion

To the best of our knowledge, our study is the first study to focus on the related impact factors of societal influences, quality of life, and investigate interrelationships between societal influences, mental health, physical health, post-traumatic stress disorder (PTSD), age, sleep score, depression score, nursing staff or not, and smoking among healthcare team members in a large psychiatric hospital. In multiple linear regressions, PTSD frequency score had positive association with SISQ score. In SEM analysis, PTSD also had positive influence on SISQ. However, sleep score and MCS value both had negative influences on SISQ. We can conclude that healthcare team members who had more severe PTSD symptoms suffered more societal influences in a psychiatric hospital under the COVID-19 pandemic. On the other hand, healthcare team members who had poor sleep and mental health quality had fewer societal influences in a psychiatric hospital during the COVID-19 pandemic. Furthermore, multiple linear regression analysis for possible related factors of social distance, social anxiety, social information, and social adaptation (four domain of SISQ) were used (Supplementary Tables 7–10). We found that PTSD frequency score had positive association with four domains of SISQ. Sleep score and MCS value only had negative association with social anxiety.

Depressive score had an indirect negative influence on SISQ by sleep score and indirect positive influence on SISQ by MCS value. We cannot understand whether the healthcare team members with higher depression score would suffer from more societal influences in a large psychiatric hospital during the COVID-19 pandemic. Then we also used ANOVA test to compare the SISQ score between four groups (Depression score = 0, 1, 2, 3, data showed at Supplementary Table 11), there was no significant statistical difference between four groups.

The major threat of the COVID-19 pandemic has seriously affected people's mental health (9, 52, 53). And healthcare team members are also under such threats, and their mental health has also been severely impacted (53, 54). Our research focused on the differences in the mental health impacts among different types of healthcare team members who suffered threats in a psychiatric hospital under the COVID-19 pandemic. We found that there is no difference in their total SISQ scores regardless of whether they are nursing staff or not, but the score of nursing staff in social anxiety was higher, while the score of nursing staff in social information was lower, which shows that nursing staff is more anxious in the face of COVID-19 pandemic, but they receive less social information. COVID-19 had more effect on the mental health of non-nursing staff than nursing staff. On the other hand, we can find that the score of nursing staff in MCS value was lower, while the score of sleep score was higher, which showed that the mental health of nursing staff was poor, and the sleep quality was worse. This may be related to the nature of the work that nursing staff required more shifts.

In the lifestyle analysis, we found that nursing staff had fewer exercise habits, less regular intake, and less participation in social activities which may be due to their working style. Most nursing staff requires shifts among their work that lead to poor lifestyle. A past study (55) showed that 70 nurses (63%) worked nightshifts within the past year and poor sleep quality was the lifestyle factor which most strongly contributed to fatigue.

We also found that the proportion of women in this group of nursing staff is relatively high, and they are younger. Some differences in scale scores, lifestyle (smoking, exercises habits, participate in social activities, regular intake), and the presence or absence of chronic diseases under *T*-test and Chi-square test may be due to differences in gender and age causing this statistically significant difference. It is possible to form Type I error, so we used multiple linear regression analysis to ascertain whether the independent factors were associated with dependent variables, including SISQ score and quality of life (MCS and PCS). And the SISQ, MCS value, PCS value, PTSD symptoms, age, sleep score, depression score, nursing staff or not, gender, education years, and smoking and other related factors were included in the structural equation model (SEM) analysis, so that Type I error caused by the use of *T*-test and Chi-square test can be avoided.

However, a past study (12) about SARS in Taiwan showed that the major difference between the mental health of the nurses and the other healthcare workers was in the somatic realm (headache, palpitations, discomfort in the chest, and numbness of the limbs) in that the nurses had fewer complaints and symptoms. Under SEM analysis in our study, nursing staff had positive influence on social anxiety. Nursing staff had positive influence on sleep score. Nursing staff had negative influence on PCS value. So we need to let our nursing staff get more social information, and nursing staff may need more psychological intervention to improve their social anxiety, sleep disturbance, and life quality during the COVID-19 pandemic in the future.

In the multiple linear regression analysis, PTSD frequency score had positive association with SISQ score and PTSD severity score had no association with SISQ score. We can conclude that relative to PTSD severity score, PTSD frequency score was more important to the societal influences among healthcare team members in a psychiatric hospital during the COVID-19 pandemic. On the other hand, older female and married members suffered from more societal influences during the COVID-19 pandemic. Healthcare team members with higher education level, regular intake suffered from more societal influences during the COVID-19 pandemic. A past study (56) showed that culture factor also had influences on social distance during the COVID-19 pandemic. But we can't consider the culture difference in a single-center study. Sleep score and MCS value only had negative effect to social anxiety, not affecting other domains of SISQ. Another study (57) showed that medical staff had higher anxiety scores and depression scores than general population and the gender, age, marriage, working years, occupation, educational level, and economic income did not affect anxiety and depression. However, we did not investigate the related questionnaires among the general population in our study.

In the multiple linear regressions, PTSD frequency score had a negative association with MCS value and PCS value of Short form-12 items health survey and PTSD severity score had no association with MCS value and PCS value. We can conclude that relative to PTSD severity score, PTSD frequency score was more important to the quality of life (physical and mental) among healthcare team members in a psychiatric hospital during the COVID-19 pandemic. However, MCS value and PCS had effects on each other which means there was a strong association between the physical component and mental component of quality life. Members with sleep problems may worsen their life quality among physical component and mental component, and a past study (58) also had a similar finding. Members with chronic illness with 1 year, smoking, and fewer exercise habits had poor life quality among physical component, like past studies (59–61). Members with older age, participation in social activities frequently had better life quality on mental component. As in past studies (62, 63), depression can worsen the life quality among mental component. Among the association between societal influences and quality of life among healthcare team members in a psychiatric hospital under COVID-19 pandemic, members with higher social distance scores had poor life quality among physical component. Members with higher social anxiety scores had poor life quality among mental component. Members with higher social information scores had better life quality among mental component.

### Limitation of the Study

First, societal influences and quality of life, other related factors like level of depression, sleep disturbance, and PTSD were measured by self-reported questionnaires in our study, and it would have been better if they had been verified through objective observations for related factors in our study. Second, the cross-sectional design of this study limited causal inference for further interpretation. A repeat measurement study may be considered to perform in the future. Finally, a single-center study may limit the generalizability and applicability to other populations.

## Conclusion

Healthcare team members who had more severe PTSD symptoms suffered more societal influences. Relative to PTSD severity score, PTSD frequency score was more important to the societal influences and quality of life among healthcare team members. On the other hand, older female and married members suffered from more societal influences. Health care team members with higher education level, regular intake suffered from more societal influences. Sleep problems may worse physical life quality and mental life quality, and depression may worse mental life quality. Members with chronic illness with 1 year, smoking, and fewer exercise habits had poor physical life quality. Members of older age, who frequently participate in social activities had better life quality among mental component.

## Data Availability Statement

The original contributions presented in the study are included in the article/Supplementary Material, further inquiries can be directed to the corresponding authors.

## Ethics Statement

The studies involving human participants were reviewed and approved by Institutional Review Board of Kai-Syuan Psychiatric Hospital. The patients/participants provided their written informed consent to participate in this study.

## Author Contributions

W-TK: conceptualization, methodology, formal analysis, investigation, writing–the main manuscript text, and preparing all figures. S-TH: formal analysis, data curation, and conceptualization. L-SC: methodology and data curation. K-YH and D-JL: methodology and investigation. G-GL, P-JW, and W-JC: investigation. FH-CC: conceptualization, methodology, formal analysis, funding acquisition, project administration, writing–review, and editing. J-JH: conceptualization, formal analysis, writing–review, and editing. All authors have read and approved the manuscript.

## Funding

This study was supported by Kaohsiung Municipal Kai-Syuan Psychiatric Hospital (KSPH) and approved by the Institutional Review Board of KSPH (KSPH-2020-03).

## Conflict of Interest

The authors declare that the research was conducted in the absence of any commercial or financial relationships that could be construed as a potential conflict of interest.

## Publisher's Note

All claims expressed in this article are solely those of the authors and do not necessarily represent those of their affiliated organizations, or those of the publisher, the editors and the reviewers. Any product that may be evaluated in this article, or claim that may be made by its manufacturer, is not guaranteed or endorsed by the publisher.

## Abbreviations

COVID-19: corona virus infection disease 2019
SF-12: Short form*-*12 *items*
SISQ: Societal Influences Survey Questionnaire
DRPST: Disaster-Related Psychological Screening Test
SEM: Structural equation model
PTSD: post-traumatic stress disorder
PCS: physical component summary
MCS: mental component summary
SARS: severe acute respiratory syndrome
MERS: Middle East respiratory syndrome
HRQoL: health-related quality of life
PSQI: Pittsburgh Sleep Quality Index.
