# Supplementary material for: The Societal Influences and Quality of Life Among Healthcare Team Members During the COVID-19 Pandemic
Source: Front Psychiatry. 2021 Oct 11;12:706443. doi: 10.3389/fpsyt.2021.706443 (PMC8542799; doi:10.3389/fpsyt.2021.706443)
Supplement: Supplementary file 1 [file Table_1.pdf]

Supplementary Table S1 Measures used in our study

Measures	Items	Response scale
Depression (DRPST[1]): In recent one month, do you have the following symptoms persisted more than two weeks?	Item 1: Depressed mood most of the day, nearly every day.	0 = no, 1 = yes
	Item 2: Fatigue or loss of energy nearly every day.	0 = no, 1 = yes
	Item 3: Feeling of worthlessness or excessive or inappropriate guilt nearly every day	0 = no, 1 = yes
Post-traumatic stress disorder, PTSD, PTSD symptoms severity (DRPST[1]): In recent one month, do you have the following symptoms during the past one month?	Item 1:Unhappy or lack of interest in most time	1=no distress, 2=little distress, 3=moderate distress, 4= significant distress, 5= extremely distress
	Item 2:It was still very difficult to relax or feel insecure without watching/thinking about COVID-19 news/events	1=no distress, 2=little distress, 3=moderate distress, 4= significant distress, 5= extremely distress
	Item 3:The body had reactions (such as palpitations, tremors, muscle tightness, sweating...) when you exposed to news/events of COVID-19 or similar situations.	1=no distress, 2=little distress, 3=moderate distress, 4= significant distress, 5= extremely distress
	Item 4:Avoiding places, activities, people or reports that remind you of the COVID-19 news/events.	1=no distress, 2=little distress, 3=moderate distress, 4= significant distress, 5= extremely distress
	Item 5:Trying to avoid thinking about the COVID-19 news/events.	1=no distress, 2=little distress, 3=moderate distress, 4= significant distress, 5= extremely distress
	Item 6:Often re-experience the image or	1=no distress, 2=little distress,

	situation of the traumatic event, as if it happened again, and feel suffering	3=moderate distress, 4= significant distress, 5= extremely distress
	Item 7:Repeated nightmare about topics related to COVID-19 news/events	1=no distress, 2=little distress, 3=moderate distress, 4= significant distress, 5= extremely distress
	Item 8:Do you feel pessimistic and uncertain about the future	1=no distress, 2=little distress, 3=moderate distress, 4= significant distress, 5= extremely distress
Post-traumatic stress disorder, PTSD, PTSD symptoms frequency (DRPST[1]): In recent one month, do you have the following symptoms during the past one month?	Item 1:Unhappy or loss of interest in most time	1=never, 2=only one time, 3=two to three times, 4=four to six times, 5=every day
	Item 2:It was still very difficult to relax or feel insecure without watching/thinking about COVID-19 news/events	1=never, 2=only one time, 3=two to three times, 4=four to six times, 5=every day
	Item 3:The body had reactions (such as palpitations, tremors, muscle tightness, sweating...) when you exposed to news/events of COVID-19 or similar situations.	1=never, 2=only one time, 3=two to three times, 4=four to six times, 5=every day
	Item 4:Avoiding places, activities, people or reports that remind you of the COVID-19 news/events.	1=never, 2=only one time, 3=two to three times, 4=four to six times, 5=every day
	Item 5:Trying to avoid thinking about the COVID-19 news/events.	1=never, 2=only one time, 3=two to three times, 4=four to six times, 5=every day
	Item 6:Often re-experience the image or situation of the traumatic event, as if it	1=never, 2=only one time, 3=two to three times, 4=four to six times,

	happened again, and feel suffering	5=every day
	Item 7:Repeated nightmare about topics related to COVID-19 news/events	1=never, 2=only one time, 3=two to three times, 4=four to six times, 5=every day
	Item 8:Do you feel pessimistic and uncertain about the future	1=never, 2=only one time, 3=two to three times, 4=four to six times, 5=every day
Sleep (PSQI[2]): During the past month,….	Item 1: Do you cannot get to sleep within 30 minutes?	1 = not during the past month, 2 = less than once a week, 3 = once or twice a week, 4 = three or more times a week.
	Item 2: Do you wake up in the middle night or early morning, and you cannot sleep again?	1 = not during the past month, 2 = less than once a week, 3 = once or twice a week, 4 = three or more times a week.
	Item 3: How would you rate your sleep quality overall?	1 = very good, 2 = fairly good, 3 = fairly bad, 4 = very bad.
	Item 4: How much of a problem has it been for you to keep up enough enthusiasm to get things done?	1 = not a problem at all, 2 = only a very slight problem, 3 = somewhat of a problem, 4 = a very big problem.
Social distance (SISQ [4]):How has COVID-19 impacted you?	Item 1: I avoid communication with or encountering strangers.	1 = never (0%), 2 = Rarely (10%), 3 = sometimes (30%), 4 = often (60% or above)
	Item 2: I avoid close or personal contact with family members and/or people I am close to.	1 = never (0%), 2 = Rarely (10%), 3 = sometimes (30%), 4 = often (60% or above)
	Item 3: I avoid going out, especially if I should require public transport	1 = never (0%), 2 = Rarely (10%), 3 = sometimes (30%), 4 = often

		(60% or above)
	Item 4: I reduce eating out.	1 = never (0%), 2 = Rarely (10%), 3 = sometimes (30%), 4 = often (60% or above)
Social anxiety (SISQ [4]): How has COVID-19 impacted you?	Item 1: I worry about the pandemic affecting my work.	1 = never (0%), 2 = Rarely (10%), 3 = sometimes (30%), 4 = often (60% or above)
	Item 2: I feel anxious or fearful due to the pandemic.	1 = never (0%), 2 = Rarely (10%), 3 = sometimes (30%), 4 = often (60% or above)
Social information (SISQ [4]): How has COVID-19 impacted you?	Item 1: I constantly check for latest pandemic news updates via television, computer or phone	1 = never (0%), 2 = Rarely (10%), 3 = sometimes (30%), 4 = often (60% or above)
	Item 2: I continuously seek out information regarding COVID-19.	1 = never (0%), 2 = Rarely (10%), 3 = sometimes (30%), 4 = often (60% or above)
Social adaptation (SISQ [4]): How has COVID-19 impacted you?	Item 1:I am more cautious of residents from severely impacted areas	1 = never (0%), 2 = Rarely (10%), 3 = sometimes (30%), 4 = often (60% or above)
	Item 2:I avoid or cancel traveling overseas (Answer "60%", if you have cancelled an overseas trip. Answer "10%" or "30%", if you are still considering your cancellation.)	1 = never (0%), 2 = Rarely (10%), 3 = sometimes (30%), 4 = often (60% or above)

[1]: DRPST=Disaster-Related Psychological Screening Test

[2]: PSQI=Pittsburgh Sleep Quality Index

[3]: SF-12v2=The 12-item Short Form Survey version 2

[4]: SISQ=Societal Influences Survey Questionnaires

Supplementary Table S2 Distribution of marital status

Marital status	n	%
Single	330	48.3
Married	309	45.2
Divorced	33	4.8
Widowed	6	0.9
Cohabited	2	0.3
Remarried	0	0
Others	3	0.4

Supplementary Table S3 Distribution of chronic illness within 1 year

Chronic illness	n	%
Hypertension	37	5.4
Dyslipidemia	20	2.9
Diabetes mellitus	20	2.9
Coronary artery disease	9	1.3
Hepato-biliary disease	8	1.2
Gastric disease	20	2.9
Lung disease	3	0.4
Cancer	6	0.9
Others	36	5.3

Supplementary Table S4. Multiple linear regression for possible related factors of Total SISQ score with 1000 bootstrapping samples

		95% CI		
Variable	Beta	Lower Bound	Upper Bound	P Value
Female	2.063	0.982	3.139	0.001
Married	0.961	0.023	1.887	0.045
Regular intake	1.721	0.575	2.884	0.006
PTSD frequency score	0.516	0.410	0.609	0.001
Age	0.066	0.017	0.111	0.005
Education years	0.254	0.048	0.445	0.005

Dependent Variable: Total score SISQ

Supplementary Table S5. Multiple linear regression for possible related factors of PCS value of SF-12 with 1000 bootstrapping samples

Variable	Beta	95% CI Lower Bound	95% CI Upper Bound	P Value
Chronic illness within 1 year	-2.381	-3.509	-1.158	0.001
Smoking	-1.833	-3.866	0.136	0.062 [a]
Exercises habits	1.730	0.843	2.628	0.001
Sleep score	-0.514	-0.713	-0.316	0.001
MCS Value	-0.092	-0.151	-0.031	0.004
PTSD frequency score	-0.306	-0.478	-0.142	0.001
Social Distance	-0.185	-0.307	-0.056	0.009

Dependent Variable: PCS value of SF-12,

[a]: excluded from bootstrapping methods

Supplementary Table S6. Multiple linear regression for possible related factors of MCS value of SF-12 with 1000 bootstrapping samples

Variable	Beta	95% CI Lower Bound	95% CI Upper Bound	P Value
Age	0.132	0.070	0.188	0.001
Participate in social activities	1.652	0.276	3.063	0.001
PTSD frequency score	-0.663	-0.846	-0.462	0.021
Sleep score	-0.749	-1.044	-0.459	0.001
Social Anxiety	-1.092	-1.540	-0.666	0.001
Social Information	0.619	0.190	1.025	0.001
Depression score	-2.804	-3.740	-1.791	0.003
PCS Value	-0.152	-0.267	-0.041	0.001

Dependent Variable: MCS value of SF-12

Supplementary Table S7. Multiple linear regression for possible related factors of Social Distance

			95% CI		
			Lower	Upper	
Variable	Beta	t	Bound	Bound	P Value
Age	0.185	5.047	0.033	0.074	<0.001
PTSD frequency score	0.163	4.479	0.074	0.190	<0.001
Female	0.154	4.212	0.577	1.583	<0.001
Regular intake	0.127	3.453	0.425	1.546	0.001

Dependent Variable: Social Distance

Supplementary Table S8. Multiple linear regression for possible related factors of Social Anxiety

Variable	Beta	t	95% CI Lower Bound	95% CI Upper Bound	P Value
PTSD frequency score	0.396	10.000	0.141	0.210	<0.001
MCS Value	-0.180	-4.433	-0.046	-0.018	<0.001
Had religion	0.102	3.030	0.125	0.584	0 .003
Nursing staff	0.086	2.534	0.066	0.519	0 .011
Sleep score	-0.080	-2.032	-0.098	-0.002	0.042

Dependent Variable: Social Anxiety

Supplementary Table S9. Multiple linear regression for possible related factors of Social Information

			95% CI		
Variable	Beta	t	Lower Bound	Upper Bound	P Value
PTSD frequency score	0.276	7.654	0.087	0.147	<0.001
Age	0.127	3.375	0.008	0.030	0.001
Had religion	0.113	3.020	0.131	0.618	0.003
Regular intake	0.089	2.436	0.070	0.651	0.015

Dependent Variable: Social Information

Supplementary Table S10. Multiple linear regression for possible related factors of Social Adaptation

			95% CI		
Variable	Beta	t	Lower Bound	Upper Bound	P Value
Education years	0.156	4.213	0.063	0.172	<0.001
PTSD frequency score	0.144	3.885	0.036	0.109	<0.001
Female	0.137	3.701	0.283	0.922	<0.001
Regular intake	0.080	2.144	0.032	0.737	0.032

Dependent Variable: Social Adaptation

Supplementary Table S11. The ANOVA test for the difference of total SISQ among healthcare team members with different depression score.

Depression score	Number	Total SISQ (Mean±SD)	F	P value
0	552	26.58±6.242	1.685	0.169
1	68	27.65±5.296		
2	29	28.55±7.424		
3	34	27.76±6.155		
